# The Impact of Ischaemic Type Biliary Lesions on Healthcare Costs After Liver Transplantation With Grafts From Donors After Circulatory Death

**DOI:** 10.3389/ti.2025.14719

**Published:** 2025-09-22

**Authors:** James M. Halle-Smith, Marta Burak, George Clarke, Angus Hann, Arul Suthananthan, Keith J. Roberts

**Affiliations:** ^1^ Liver Unit, Queen Elizabeth Hospital Birmingham, Birmingham, United Kingdom; ^2^ College of Medical and Dental Sciences, University of Brimingham, Birmingham, United Kingdom

**Keywords:** donation after circulatory death (DCD), biliary stricture, machine perfusion, costs and benefits, liver transplant

Dear Editors,

Ischaemic type biliary lesions (ITBL) are characterised by diffuse, nonanastomotic intrahepatic biliary strictures with upstream dilatation, in the absence of other complications such as hepatic artery stenosis or ductopenic rejection [1, 2]. They are identified most commonly through magnetic resonance cholangiopancreatography (MRCP) [3, 4]. The resulting cholestasis leads to recurrent infections, the need for repeated biliary drainage procedures and eventually a large proportion will suffer graft loss [3,4].

The exact aetiology of ITBL is still not known, however a greater rate of ITBL has been reported grafts from donors after circulatory death (DCD) [5–8], likely due to ischaemia/reperfusion injury, microvascular thromboses and/or cytotoxic injury [3, 4].

Efforts to reduce waiting lists for liver transplantation have led to increasing use of marginal grafts, including an increased use of those from DCD donors. Furthermore, a marked increase in the number of DCD donors has been observed [9], with recent national figures showing that DCD donors now make up close to half of all donors [10]. This brings the need for optimisation of these grafts to the forefront.

There is evidence to suggest that novel perfusion and preservation strategies such as normothermic regional perfusion (NRP) and hypothermic oxygenated machine perfusion (HOPE) can reduce rates of biliary complications in DCD donors [11, 12]. However, these incur extra costs to health service providers who will understandably seek reassurance that the extra expenditure required to fund these technologies is justified.

We aimed to investigate the long-term impact of ITBL on health service utility at our institution after liver transplantation with DCD grafts and in so doing propose potential savings with new technology.

To ensure long-term follow-up consecutive whole static cold storage (SCS) DCD liver transplants between 2016 and 2018 were reviewed from our prospectively maintained institutional database. To be classified as having ITBL, patients required a diagnostic magnetic resonance cholangiopancreatography (MRCP) scan and the absence of anastomotic stricture. Those who underwent liver transplantation for primary sclerosing cholangitis (PSC) were excluded due to difficulties in distinguishing recurrent PSC from ITBL on MRCP. Those with concurrent hepatic artery stenosis or thrombosis were also excluded. To compare healthcare costs between patients who developed ITBL and the standard DCD cohort, ITBL patients were matched to patients who received a DCD SCS graft during the same period and did not develop ITBL. Matching was based on age (+/− 5 years), indication for transplant and UKELD (+/- 5) at the time of listing. For ITBL and matched patients, all hospital episodes after discharge from index transplant were retrieved using the electronic hospital record. Cost codes for each procedure or episode were obtained from the latest available NHS tariffs (2022/23). Graft and patient survival was calculated from the date of transplant to the date of death, retransplantation or last follow-up.

Of 115 DCD liver transplants during the study period, 19 developed ITBL (16.5%). Graft survival was significantly lower in the ITBL group (23.4 months vs. 72.8 months; p = 0.001), with 10 (53%) of the patients requiring retransplantation.

The total hospital costs were significantly higher amongst the ITBL group, with an average cost per patient of £111,675.80 (Range: £3,116-£271,278) compared to £17,817.11 (Range: £3,982 - £93,171) in the matched “No ITBL” control group (Table 1). A large contributor to the increased cost was retransplantation, however significantly increased costs due to increased use of diagnostic imaging and procedures, such as biopsies, were also observed. In addition, the ITBL group had a markedly increased number of readmission bed days (1006 days vs. 66 days; p = 0.002) (Table 1).

**TABLE 1:** Comparison of healthcare costs after index liver transplantation between ITBL and no ITBL controls.

Tertiary centre hospital episode		Unit cost	NHS tariff code	No ITBL (n = 19)		ITBL (n = 19)		p-value
				N	Mean cost per pt	N	Mean cost per pt	
Subsequent Operative Procedures								
	Retransplant	£80,000	N/A	0	£0	10	£42,105.26	**0.012**
	Incisional hernia repair	£6,760	FF60A	3	£1,067	0	£0.00	0.418
	Hepaticojejunostomy	£21,495	GA03C	0	£0	1	£1,131.32	0.795
	Laparotomy and washout	£21,495	GA03C	1	£1,131	3	£3,393.95	0.583
Interventional Radiology								
	ERCP	£9,653	GB09D	4	£2,032	20	£10,161.05	0.234
	PTC drainage/imaging	£1,830	YG06Z	5	£482	4	£385.26	1
	TIPPS with stent	£5,274	YA10Z	0	£0	1	£277.58	0.795
	Angiogram+/-stenting	£5,274	YA10Z	2	£555	1	£277.58	1
	Hepatic venogram	£5,274	YA10Z	1	£278	6	£1,665.47	1
	CT guided drain	£10,005	YF04A	2	£1,053	4	£2,106.32	0.603
	US guided drain	£10,005	YF04A	4	£2,106	19	£10,005.00	0.402
	Fluoroscopic guided drain	£10,005	YF04A	0	£0	2	£1,053.16	0.795
	CT liver ablation	£7,563	YG01A	1	£398	0	£0.00	0.795
Diagnostic Radiology								
	CT	£95	RD24Z	28	£140	73	£365.00	0.146
	MRI Liver	£178	RD03Z	3	£28	8	£74.95	0.37
	MRCP	£116	RD01A	11	£67	46	£280.84	**<0.001**
	US abdomen	£55	RD42Z	105	£304	205	£593.42	**0.006**
	US guided biopsy	£907	YF05Z	5	£239	22	£1,050.21	**0.043**
	NM	£1,045	YG12Z	0	£0	2	£110.00	0.583
	CXR	£28	N/A	74	£109	164	£241.68	0.085
	AXR	£28	N/A	0	£0	9	£13.26	0.172
	PICC	£1,729	YR42A	2	£182	13	£1,183.00	0.37
	Transjugular biopsy	£1,676	YG10Z	1	£88	4	£352.84	1
	Tubogram	£1,045	YG12Z	0	£0	2	£110.00	0.795
Follow-up								
	OPA	£206	306	622	£7,114	650	£7,417.37	1
	Readmission ITU Bed Days	£2,737	N/A	5	£720.26	67	£9,651.53	
	Readmission Ward Bed Days	£397	N/A	61	£1,274.58	939	£19,620.16	**0.002**
Total cost								
		-	-	-	£17,817.11	-	£111,675.80	**0.007**

Bold values indicates the significant at the p < 0.05 level.

This cost-utility analysis demonstrates that the development of ITBL after DCD liver transplantation leads to significantly increased healthcare costs compared to matched “No ITBL” controls. Whilst decisions to fund novel perfusion and preservation technologies are complex, these findings show that ITBL represent a significant cost burden to the health service after liver transplantation and should be considered in future funding decisions.

The evidence for the efficacy of novel perfusion and preservation strategies in reducing non-anastomotic strictures (NAS) in DCD grafts is growing. NRP, which involves restoring circulation of warm, oxygenated blood in a controlled DCD setting [13, 14], has been shown to reduce ischaemic cholangiopathy [13], with two recent meta-analysis showing that NRP significantly reduces the NAS rate in DCD grafts compared to SCS [12, 15]. Recent evidence, including a randomised controlled trial, has shown that HOPE also reduces the risk of NAS after DCD donation compared to static cold storage [16–18]. Given that this study demonstrates a significant increase in follow-up and treatment costs for patients that develop ITBL, it follows that any novel perfusion and preservation strategies that reduce ITBL after DCD liver transplantation will significantly reduce follow-up costs.

These technologies will also have an impact upon waiting lists, which continue to grow [19]. From the aspect of donation, the latest figures from the United Kingdom (UK) show a decrease of 2% in the number donors after brain death (DBD) whilst the number of DCD donors has increased by 7% [19]. It is therefore incumbent upon the liver transplantation community to expand the use of DCD grafts in a safe manner. From the recipient side, through a reduction in ITBL, fewer grafts will be required for retransplantation.

Decisions of whether to fund new technologies to optimise DCD grafts, such as NRP and HOPE, are complex and this study only looks at one aspect. For example, in establishing a new service there are training, staff and consumable costs that must be accounted for. However, evidence that novel perfusion and strategies such as NRP and HOPE can both improve outcomes of DCD grafts and increase the number of DCD grafts that can be used safely continues to grow. Health service providers must therefore now weigh up the costs of growing waiting lists and complications, which are more prevalent with DCD SCS grafts, and compare these to the costs of introducing novel perfusion and preservation strategies for DCD grafts more widely.

## Data Availability

The original contributions presented in the study are included in the article/supplementary material, further inquiries can be directed to the corresponding author.
